# Supplementary material for: 3D genomic analysis reveals novel enhancer-hijacking caused by complex structural alterations that drive oncogene overexpression
Source: Nat Commun. 2024 Jul 20;15:6130. doi: 10.1038/s41467-024-50387-w (PMC11271278; doi:10.1038/s41467-024-50387-w)
Supplement: Supplementary file 1 — Supplementary Information [file 41467_2024_50387_MOESM1_ESM.pdf]

3D genomic analysis reveals novel enhancer-hijacking caused by complex structural alterations that drive oncogene overexpression

Mortenson et al.

Supplementary Information

Included are 9 Supplementary Figures (including figure legends) and 3 Supplementary Tables.

Supplementary Figure 1

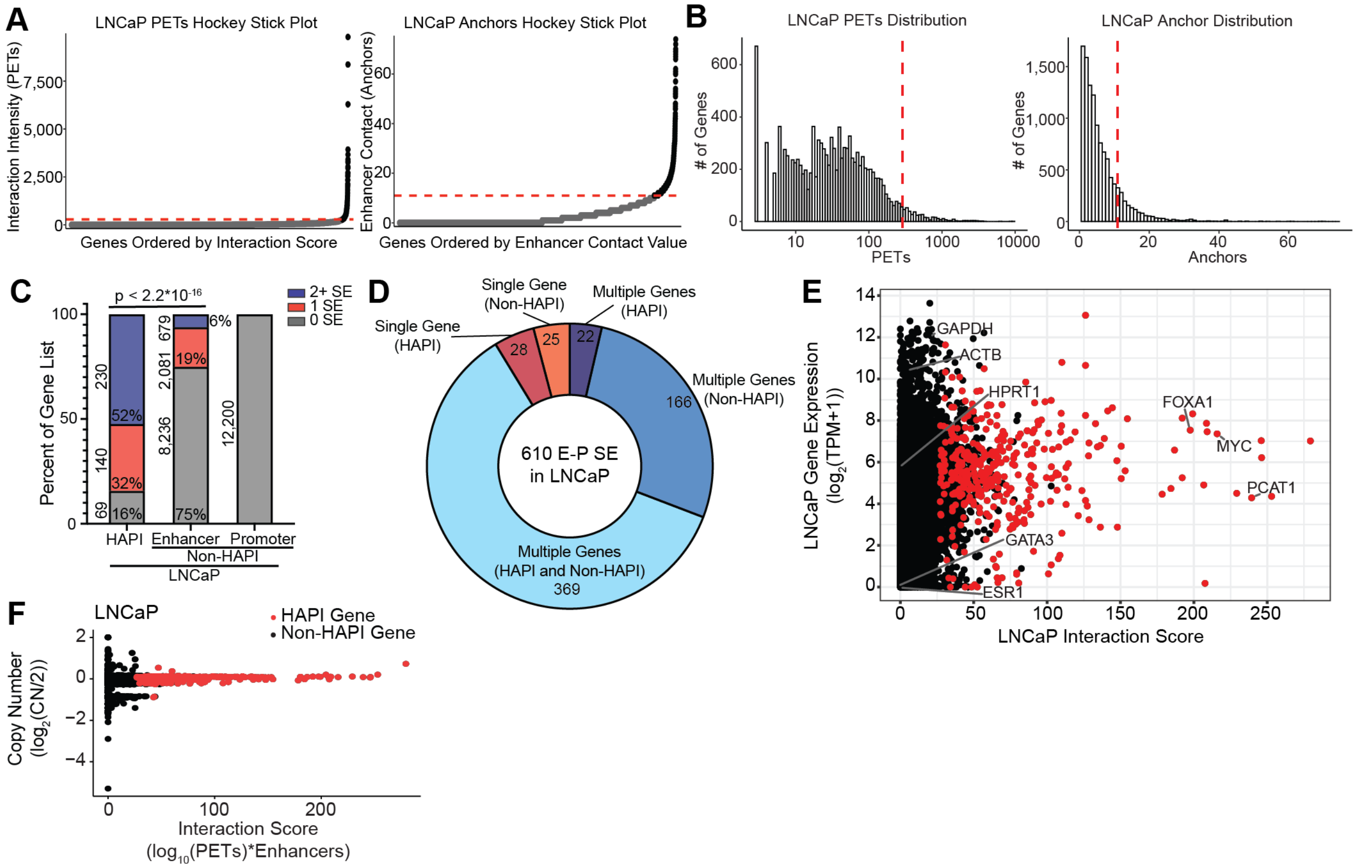

Figure S1: Additional profiling of HAPI genes. A: Hockey stick plots showing LNCaP interaction intensity and enhancer contact values of each gene in LNCaP cells, with inflection point cutoffs specified (red dashed lines). **B:** Distribution of interaction intensity and enhancer contact values for all genes in LNCaP with cutoffs from inflection points specified (red dashed lines). **C:** The percentage of HAPI genes and enhancer-connected or promoter-driven (no enhancer interaction) non-HAPI genes that are looped to super-enhancers. P-value was calculated using two-sided Fisher exact tests. **D:** The percentage of super-enhancers, involving enhancer-promoter (E-P) loops, that are looped to HAPI and/or non-HAPI genes in LNCaP cells. **E:** Interaction score versus log2(TPM+1) gene expression for HAPI genes (red) and non-HAPI genes (black) in LNCaP. **F:** Interaction score versus copy number (log2(CN/2)) for HAPI genes (red) and non-HAPI genes (black) in LNCaP.

Supplementary Figure 2

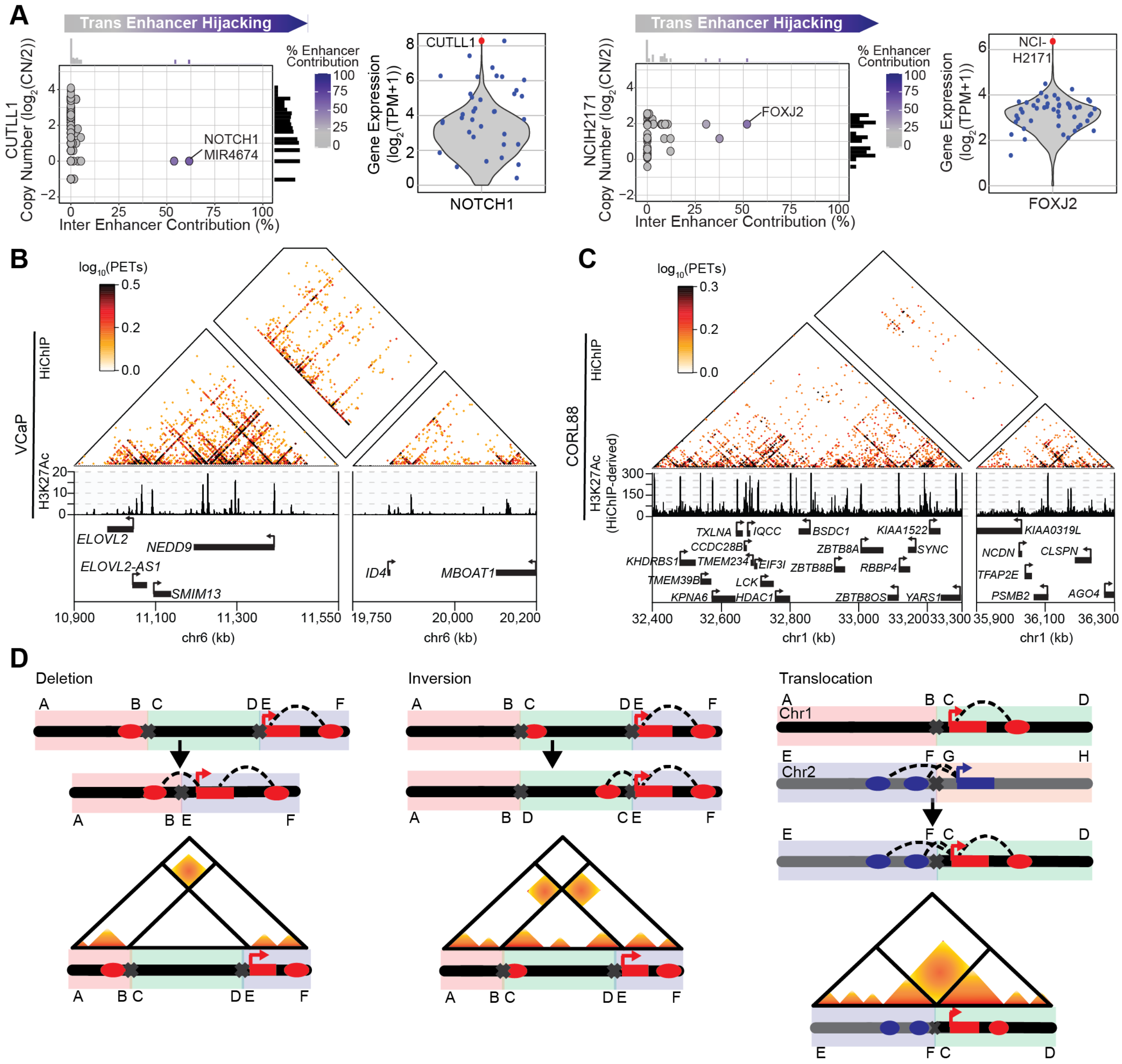

Figure S2: Additional examples of enhancer-hijacking genes identified by HAPI analysis. A: Inter enhancer contribution and copy number distribution of all HAPI genes for CUTLL1 and NCIH2171 with trans enhancer hijacking genes *NOTCH1* and *FOXJ2* (blue) labeled. Gene expression plots for the enhancer hijacking genes *NOTCH1* and *FOXJ2* generated using RNA-seq data with the identified cell lines CUTLL1 and NCIH2171 (red dots), respectively, compared to all cell lines of the same cancer type (blue dots) and all CCLE cell lines (grey violin plots). **B-C:** H3K27ac HiChIP and ChIP-seq signal at chromosomes 6 and 1 regions in VCaP (B) and CORL88 (C), respectively. The H3K27ac ChIP-seq signal in CORL88 is derived from HiChIP reads. **D:** Schematic showing different types of structural alterations, the resulting enhancer-promoter interactions, and how these structural variants would appear in HiChIP heatmaps.

Supplementary Figure 3

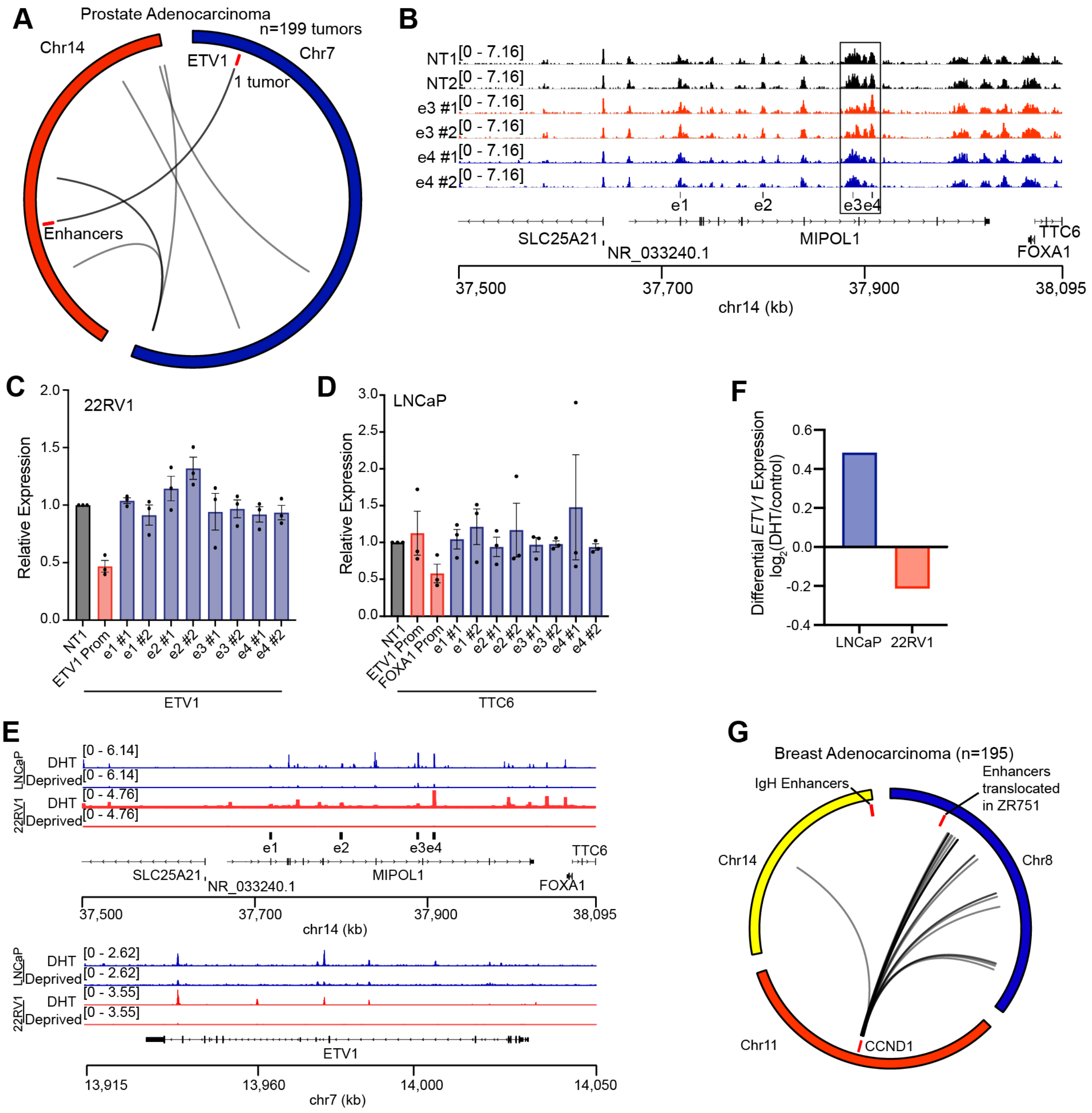

Figure S3: Additional characterization of enhancer hijacking in LNCaP and tumor samples. A: Circos plot displaying translocations between chromosomes 7 and 14 in 199 prostate adenocarcinoma tumor samples from PCAWG data set with *ETV1* and identified hijacked enhancers annotated. **B:** H3K27ac ChIP-seq in LNCaP-dCas9-KRAB-MeCP2 cells infected with non-targeting sgRNAs (NT1 and NT2) and enhancer targeting sgRNAs (e3 and e4). **C-D:** RT-qPCR measuring expression changes of *ETV1* in 22RV1 (C) and *TTC6* in LNCaP (D) after CRISPRi of each defined enhancer e1-e4 or the promoters of *ETV1* and *FOXA1/TTC6*. Gene expression levels were normalized to cells treated with the non-targeting sgRNA NT1. n = 3 biologically independent experiments. Data are presented as mean values +/- SEM. Source data are provided as a Source Data file. **E:** AR ChIP-seq in LNCaP and 22RV1 cells with and without DHT treatments for genomic regions containing the hijacked enhancers (top) and *ETV1* (bottom). **F:** log2-transformed fold changes of RNA-seq counts per million values for *ETV1* in LNCaP and 22RV1 cells with and without DHT treatments. **G:** Circos plot displaying translocations among chromosome 8, 11, and 14 (lines for translocations stemming from a 2 Mb window centered at *CCND1*) in 195 breast adenocarcinoma tumors samples from PCAWG data set with *CCND1* and identified hijacked enhancers annotated.

Supplementary Figure 4

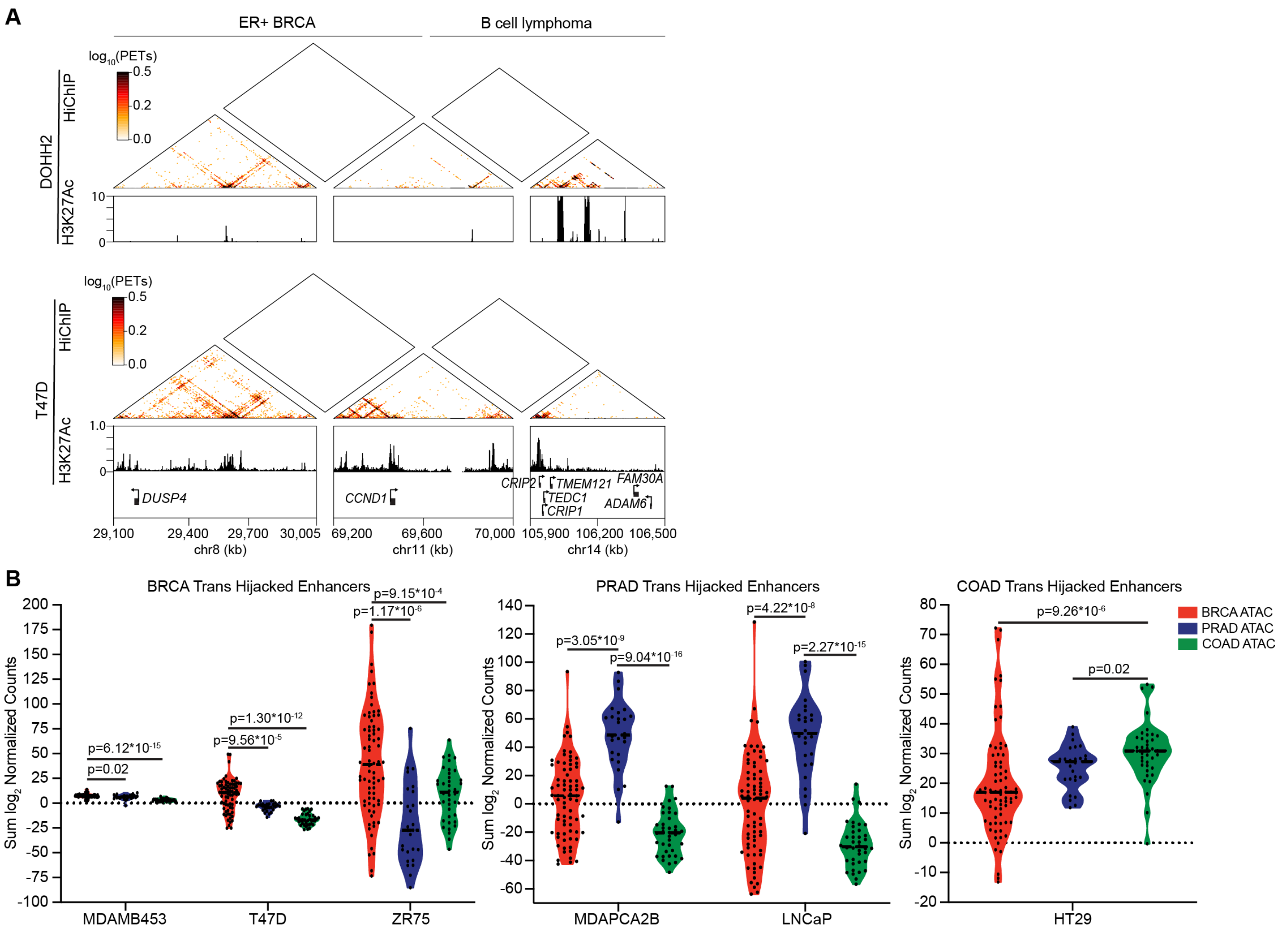

Figure S4: Enhancer origin of hijacked enhancers is cancer-type specific. A: DOHH2 and T47D H3K27ac HiChIP and ChIP-seq signal at the *CCND1* locus and the hijacked enhancer regions found in REC1 and ZR751 cells. **B:** Comparison of log2 normalized counts of the TCGA ATAC-seq peaks, overlapping with HAPI gene-hijacked enhancers in the indicated cell lines, for breast cancer (BRCA), prostate cancer (PRAD), and colorectal cancer (COAD) TCGA tumor tissues. P-values were calculated from two-sided wilcox tests.

Supplementary Figure 5

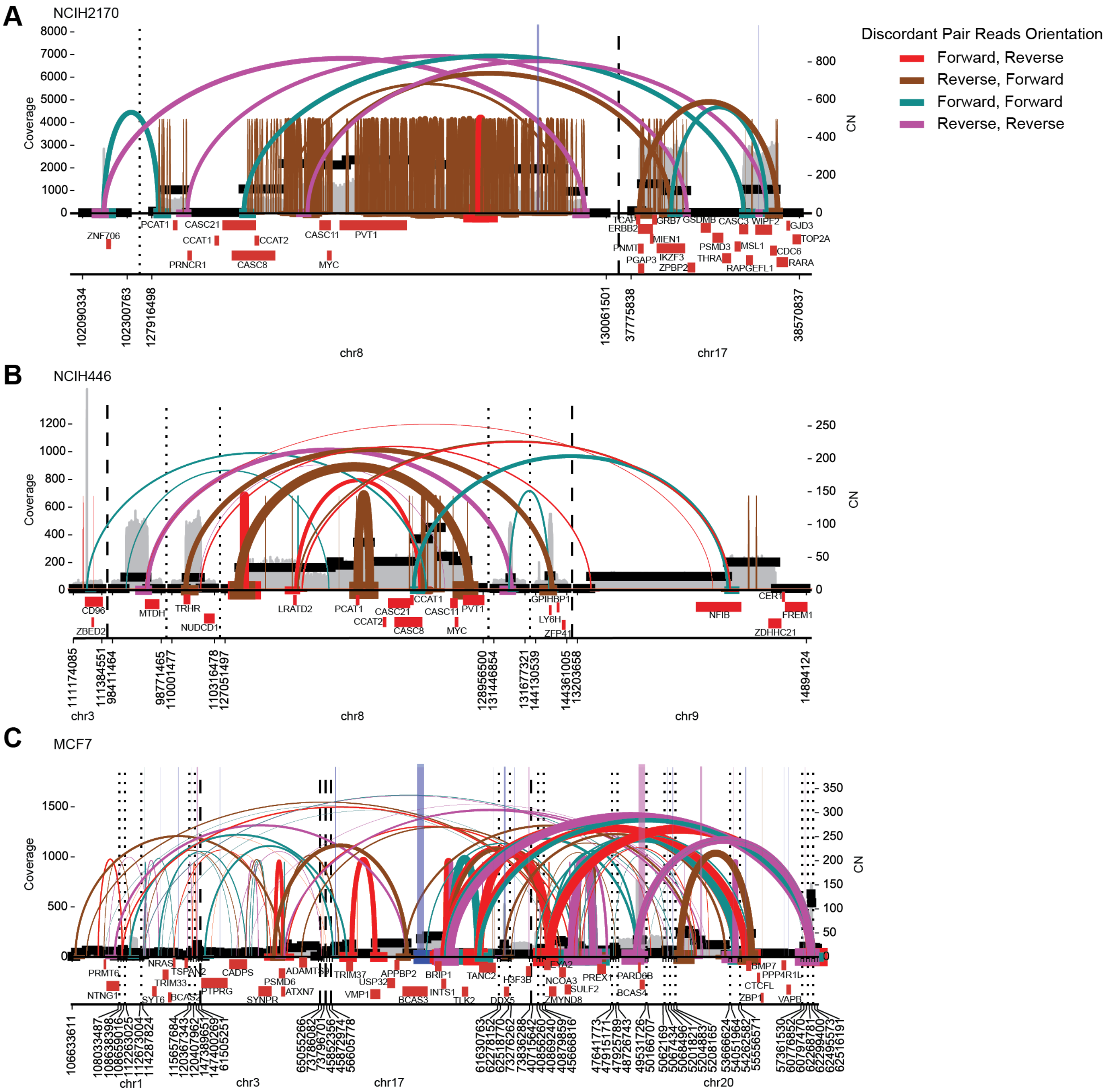

Figure S5: AmpliconArchitect reconstructions for NCIH2170, NCIH446, and MCF7. A-C: AmpliconArchitect plots depicting WGS-derived copy number and structural variants with labeled gene tracks for NCIH2170 (A), NCIH446 (B), and MCF7 (C).

Supplementary Figure 6

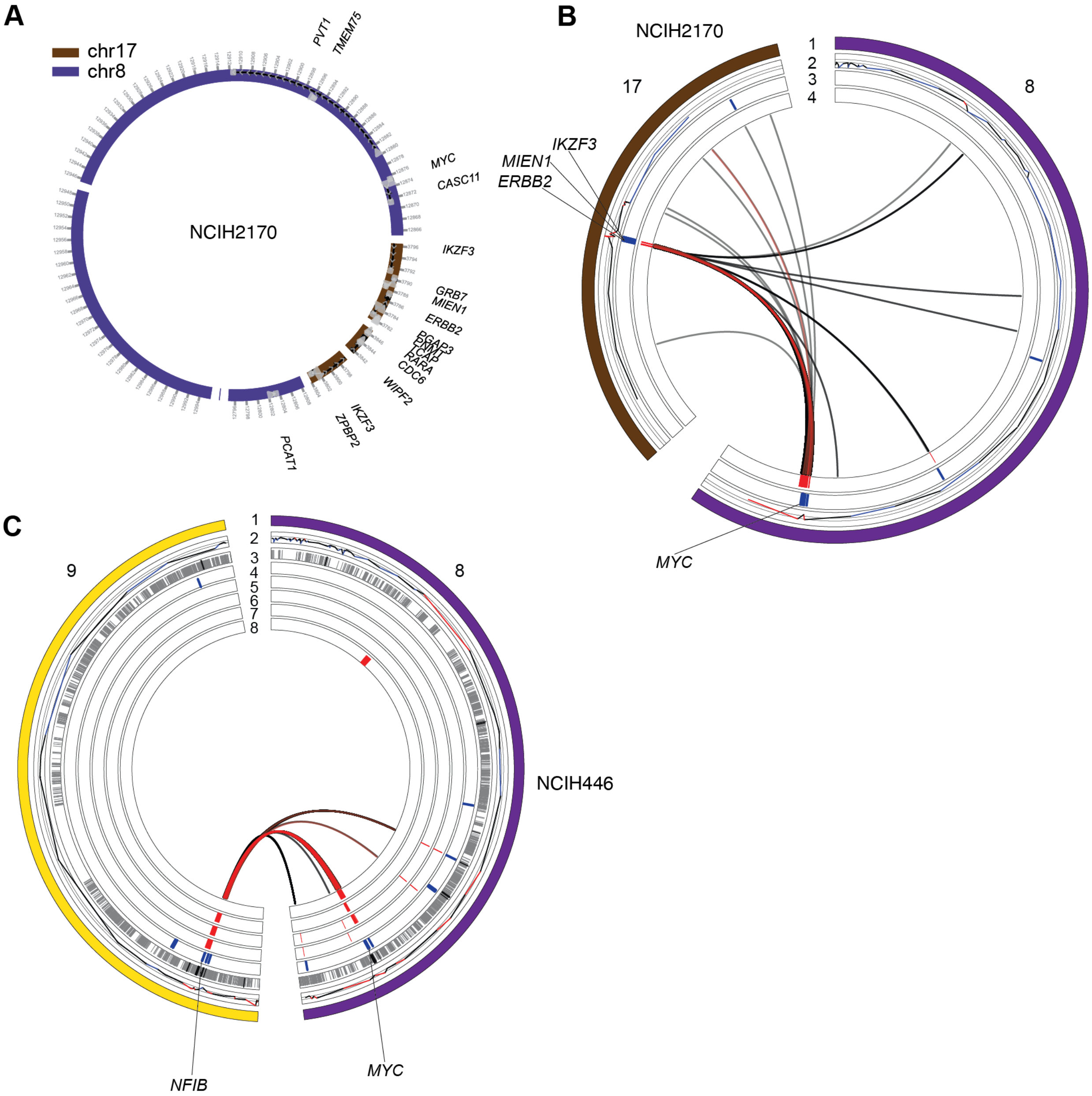

Figure S6: Complex amplicons in NCIH2170 and NCIH446. A: Structure of a proposed ecDNA in NCIH2170 based on AmpliconArchitect results. **B:** Circos plot of chromosomes 17 and 8 in NCIH2170 showing: 1) chromosome, 2) copy number, 3) TSS of HAPI genes in NCI2170, 4) DNA segments associated with the potential ecDNA, and in the center HiChIP enhancer-promoter interactions of HAPI genes (black), and WGS translocations (red). **C:** Circos plot of chromosomes 9 and 8 in NCIH446 showing: 1) chromosome, 2) copy number, 3) H3K27ac peaks showing enhancers (grey) and super-enhancers (black), 4) TSS of HAPI genes in NCIH446, 5-8) DNA segments of AmpliconArchitect-predicted amplicons, and in the center HiChIP enhancer-promoter interactions of HAPI genes (black), and WGS translocations (red).

Supplementary Figure 7

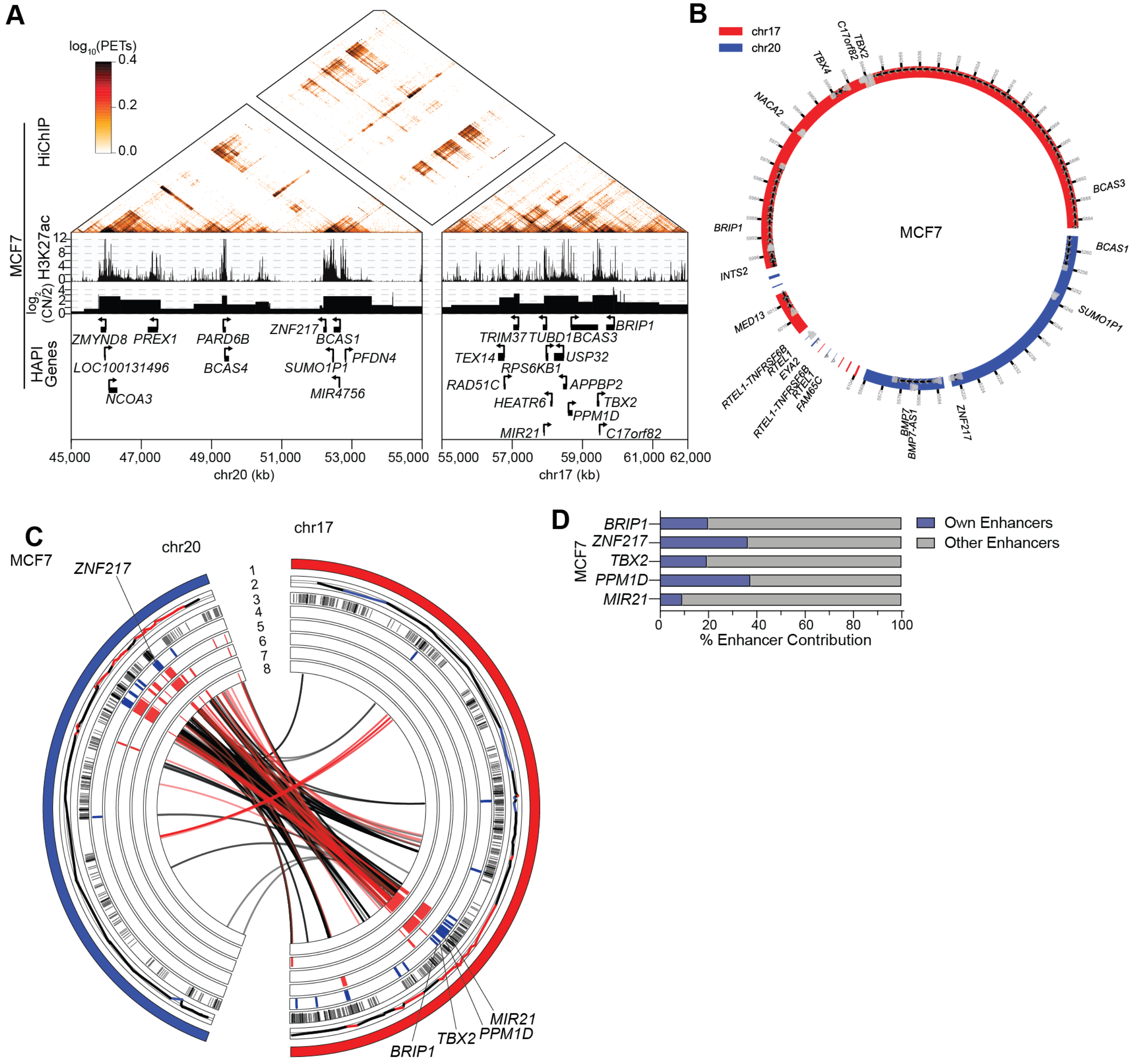

Figure S7: A potential chimeric ecDNA event in MCF7. A: H3K27ac HiChIP heatmap showing inter-chromosomal interactions between regions of chromosomes 17 and 20. **B:** Structure of a proposed ecDNA in MCF7 based on the AmpliconArchitect results. **C:** Circos plot of chromosomes 20 and 17 in MCF7 showing 1) chromosome, 2) copy number, 3) H3K27ac peaks showing enhancers (grey) and super-enhancers (black), 4) TSS of HAPI genes in MCF7, 5-8) DNA segments of AmpliconArchitect-predicted complex amplicons, and in the center HiChIP enhancer-promoter interactions of HAPI genes (black), and WGS translocations (red). **D:** Enhancer distribution of oncogenes harbored in the predicted complex amplicons in MCF7 cells. Enhancers within 2mb to a genes' promoter in the endogenous chromosomal locus will be considered as the gene's 'own' enhancers, otherwise will be considered as 'other' enhancers.

Supplementary Figure 8

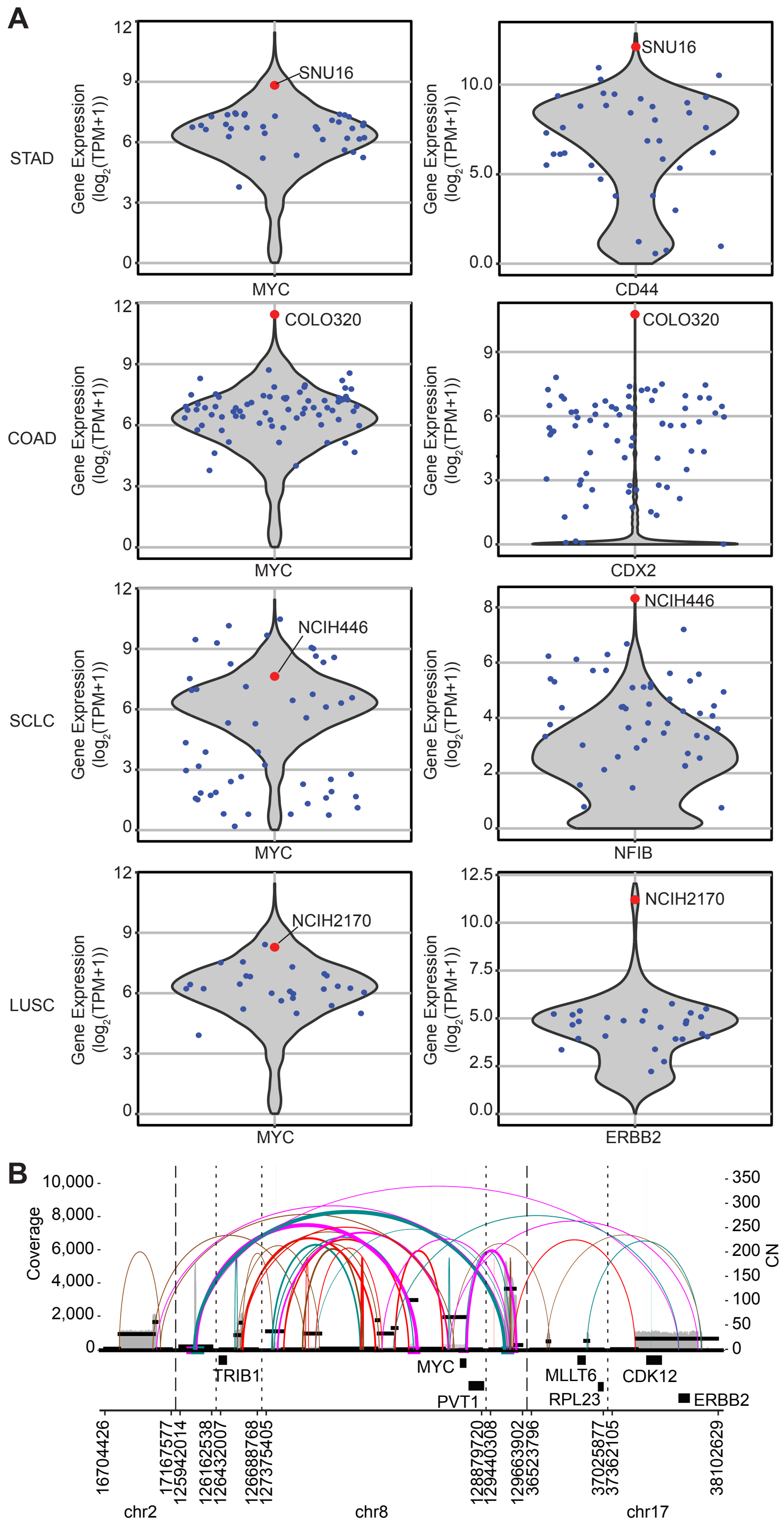

Figure S8: Gene expression of ecDNA genes in cell lines of interest and profiling ecDNA in a PCAWG sample. A: Expression of selected cancer-related genes present on ecDNAs in cell lines (red) representing STAD, COAD, SCLC, or LUSC compared to all other cell lines of the given cancer type (blue) and all cell lines in CCLE (grey violin plots). **B:** AmpliconArchitect reconstruction depicting WGS-derived copy number and structural variants for a PCAWG tumor sample containing the oncogenes *ERBB2* and *MYC*, similar to the structure found in NCIH2170.

Supplementary Figure 9

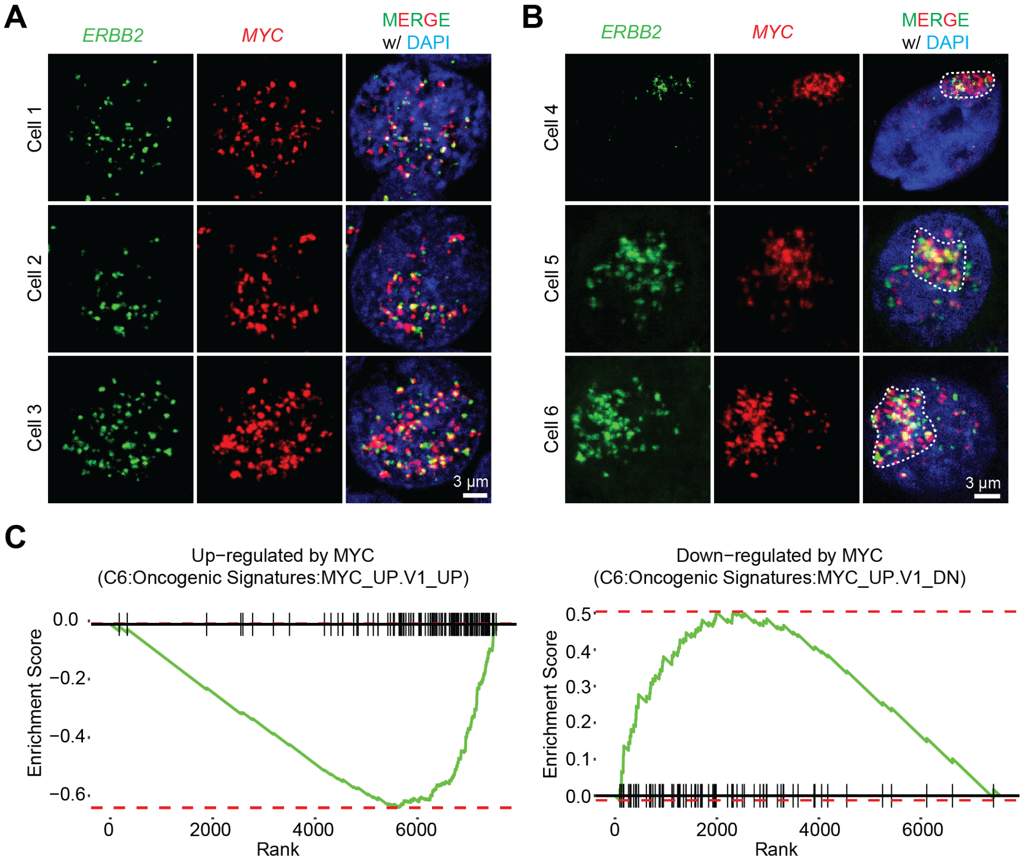

Figure S9: Interphase DNA FISH imaging shows the spatial distribution of chimeric ecDNA. A: Representative images of NCIH2170 cells in interphase showing the colocalization of *MYC* (red) and *ERBB2* (green) within cell nuclei (blue). In total, 219 interphase nuclei were collected from 8 separate images, which indicate the presence of high abundant *MYC-ERBB2* chimeric ecDNAs. **B:** The *MYC-ERBB2* ecDNAs can cluster into spatially segregated hubs in NCIH2170 cells. **C:** GSEA analysis measuring differential gene expression (CRISPRi *MYC* promoter vs non-targeting) of MYC up-regulated (left) and down-regulated (right) genes.

Supplementary Table 1: Cell lines and their associated omics data used in the study

Cell Line	HiChIP Data	HiC-Pro allValidPairs	FASTQ File	Anchor Calling	H3K27ac ChIP-seq Data	WGS Data	AR ChIP-seq Data
LNCaP	GSE227242	37075641	merged	broadpeak	GSE73994	PRJNA361316	GSE174109
T47D	GSE227242	37390012	single	broadpeak	GSE63109	N/A	N/A
CUTLL1	GSE228247	39244038	merged	Self	N/A	N/A	N/A
REC1	GSE228247	32423683	merged	Self	GSE69558	N/A	N/A
VCaP	GSE228247	20076305	single	broadpeak	GSE105760	N/A	N/A
MDAPCA2B	GSE228247	45838476	merged	Self	N/A	N/A	N/A
22RV1	GSE228247	20175335	single	broadpeak	GSE105627	N/A	GSE94013
ZR751	GSE228247	51263859	merged	broadpeak	GSE85158	PRJNA523380	N/A
MCF7	GSE228247	59028122	merged	broadpeak	GSE57436	PRJNA523380	N/A
MDAMB231	GSE97585	37514192	single	Self	N/A	N/A	N/A
MDAMB453	GSE157381	37821066	single	Self	N/A	N/A	N/A
DOHH2	GSE228247	41769569	merged	Self	GSE86743	N/A	N/A
HT29	GSE228247	26395716	single	broadpeak	GSE73319	N/A	N/A
HT55	GSE147854	19478944	single	Self	N/A	N/A	N/A
LK2	GSE166232	13705192	single	Self	N/A	N/A	N/A
H520	GSE166232	20709304	single	Self	N/A	N/A	N/A
DMS273	GSE151002	36476957	merged	Self	N/A	N/A	N/A
H69	GSE151002	23465687	single	Self	N/A	N/A	N/A
H146	GSE151002	35660665	merged	Self	N/A	N/A	N/A
H446	GSE151002	39535685	merged	Self	GSE115123	PRJNA523380	N/A
H2170	GSE228247	37495342	merged	Self	N/A	PRJNA523380	N/A
SHP77	GSE151002	21552164	single	Self	N/A	N/A	N/A
COLO320DM	GSE159985	13880280	single	Self	GSE159972	PRJNA506071	N/A
SNU16	GSE159985	103392115	single	Self	GSE159972	PRJNA523380	N/A
HMEC	GSE188401	35395988	single	Self	N/A	N/A	N/A
PrEC	GSE188401	56483476	single	Self	N/A	N/A	N/A
CORL88	GSE151002	17946661	single	Self	N/A	N/A	N/A
CORL279	GSE151002	17010536	single	Self	N/A	N/A	N/A
H524	GSE151002	12298085	single	Self	N/A	N/A	N/A
H1105	GSE151002	18409434	single	Self	N/A	N/A	N/A
H1694	GSE151002	19689472	single	Self	N/A	N/A	N/A
H1836	GSE151002	19539922	single	Self	N/A	N/A	N/A
H1876	GSE228247	52514472	merged	Self	N/A	N/A	N/A
H2029	GSE151002	27050233	single	Self	N/A	N/A	N/A
H2171	GSE151002	18707959	single	Self	N/A	N/A	N/A
H2066	GSE151002	19674767	single	Self	N/A	N/A	N/A

Supplementary Table 2: Pathway analysis for shared HAPI genes in each cancer type listed in Figure 1D (Note: only the top ranked pathway is listed in each category.)

Cluster Groups	KEGG Legacy Pathways		REACTOME		Gene Ontology Molecular Function	
	Pathway	FDR	Pathway	FDR	Pathway	FDR
Blood	B-cell receptor signaling pathway	$4.74e^{-5}$	Hemostasis	$2.53e^{-7}$	Transcription regulatory activity	$6.81e^{-17}$
BRCA	Systemic lupus erythematosus	$9.33e^{-13}$	Signaling by Nuclear Receptors	$8.49e^{-21}$	Transcription regulatory activity	$4.6e^{-20}$
COAD	Pathways in cancer	$3.26e^{-11}$	Developmental Biology	$2e^{-11}$	Transcription regulatory activity	$4.39e^{-12}$
LUSC	Pathways in cancer	$2.75e^{-6}$	Developmental Biology	$5.73e^{-7}$	Transcription regulatory activity	$4.01e^{-15}$
PRAD	Systemic lupus erythematosus	$9.91e^{-12}$	Signaling by Nuclear Receptors	$2.51e^{-20}$	Transcription regulatory activity	$4.38e^{-26}$
SCLC	Cell cycle	$2.85e^{-4}$	Diseases of signal transduction by growth factor receptors and second messengers	$2.17e^{-6}$	Transcription regulatory activity	$1.1e^{-12}$
STAD	MAPK signaling pathway	$7.42e^{-4}$	Nuclear Events (kinase and transcription factor activation)	$3.63e^{-3}$	DNA binding transcription factor binding	$1.08e^{-10}$

Supplementary Table 3: Primers used in the study

sgRNAs for CRISPRi

Name	Sequence	Blat Regions (hg19)	Notes
sg_NT1_F	CACCGCTGAGTGAAAAATAAAAGTT	N/A	Negative Control
sg_NT1_R	AAACAACTTTTATTTTTCACTCAGC		
sg_NT2_F	CACCGATCGTTTCCGCTTAACGGCG	N/A	Negative Control
sg_NT2_R	AAACCGCCGTTAAGCGGAAACGATC		
sg_ETV1_promoter_F	CACCGGGTCAGCAATAAACAACAA	chr7:14031070-14031090	
sg_ETV1_promoter_R	AAACTTGTTGTTTATTGCTGACCC		
sg_FOXA1_promoter_F	CACCGAGGGGACAATGAAGAGAAAC	chr14:38063490-38063509	
sg_FOXA1_promoter_R	AAACGTTTCTCTTCATTGTCCCCTC		
sg_LNCaP_e1_1_F	CACCGGAGAACTGGTCAAAAGGTA	chr14:37717096-37717115	
sg_LNCaP_e1_1_R	AAACTACCTTTTGACCAGTTCTCC		
sg_LNCaP_e1_2_F	CACCGAGGTATTAATACCAAGCCCC	chr14:37717126-37717145	
sg_LNCaP_e1_2_R	AAACGGGGCTTGGTATTAATACCTC		
sg_LNCaP_e2_1_F	CACCGCTTAATAAACTATTAAACTG	chr14:37798906-37798926	
sg_LNCaP_e2_1_R	AAACCAGTTTAATAGTTTATTAAGC		
sg_LNCaP_e2_2_F	CACCGTAAAACACAAAGCAGAGTTC	chr14:37798962-37798981	
sg_LNCaP_e2_2_R	AAACGAACTCTGCTTTGTGTTTTAC		
sg_LNCaP_e3_1_F	CACCGTTTCCTCCTCTATAAAAGTA	chr14:37887169-37887188	
sg_LNCaP_e3_1_R	AAACTACTTTTATAGAGGAGGAAAC		
sg_LNCaP_e3_2_F	CACCGCCAAGACCATGAATGGAAAT	chr14:37887324-37887343	
sg_LNCaP_e3_2_R	AAACATTTCCATTCATGGTCTTGGC		
sg_LNCaP_e4_1_F	CACCGCCCAGTCTTAAAAATACTGA	chr14:37905889-37905908	
sg_LNCaP_e4_1_R	AAACTCAGTATTTTTAAGACTGGGC		
sg_LNCaP_e4_2_F	CACCGAAAATAACAATGTTAAGTCC	chr14:37905937-37905956	
sg_LNCaP_e4_2_R	AAACGGACTTAACATTGTTATTTTC		
sg_CCND1_promoter_F	CACCGCGAGGGGCAGAAGAGCGCGA	chr11:69455974-69455993	
sg_CCND1_promoter_R	AAACTCGCGCTCTTCTGCCCCTCGC		
sg_ZR75_e1_F	CACCGCTCATGGAGAAAAGGAACAA	chr8:29380258-29380277	
sg_ZR75_e1_R	AAACTTGTTCCTTTTCTCCATGAGC		
sg_ZR75_e2_F	CACCGTAACAGCTTAAAGGTTATTG	chr8:29387600-29387619	
sg_ZR75_e2_R	AAACCAATAACCTTTAAGCTGTTAC		
sg_ZR75_e3_F	CACCGACCTGGTAAAGTTCAACTGA	chr8:29596128-29596147	
sg_ZR75_e3_R	AAACTCAGTTGAACTTTACCAGGTC		
sg_ZR75_e4_F	CACCGCAGGAATTTCTTATTTGCAT	chr8:29614509-29614528	
sg_ZR75_e4_R	AAACATGCAAATAAGAAATTCCTGC		
sg_ZR75_e5_F	CACCGGACAGGTGACCCTGCCTGC	chr8:29631787-29631806	
sg_ZR75_e5_R	AAACGCAGGCAGGGTCACCTGTCC		
sg_MYC_promoter_sg1_F	CACCGTAATTCCAGCGAGAGGCAG	chr8:128748488-128748507	
sg_MYC_promoter_sg1_R	AAACCTGCCTCTCGCTGGAATTAC		
sg_MYC_promoter_sg2_F	CACCGCAGCGCAGCTCTGCTCGCC	chr8:128748546-128,748565	
sg_MYC_promoter_sg2_R	AAACGGCGAGCAGAGCTGCGCTGC		

RT-qPCR Primers

Name	Sequence	Target Gene	Notes
RT_CTCF_F	CCCACACCGGGGAGAAGCCT	CTCF	Loading control
RT_CTCF_R	CGCCATCTGGGCCAGCACAA		
RT_ETV1_F	CTTAGCCGTTCACTCCGCTA	ETV1	
RT_ETV1_R	TCCTCCTCGTTGATGTGACG		
RT_CCND1_F	TACTACCGCCTCACACGCTT	CCND1	
RT_CCND1_R	CTTGGGGTCCATGTTCTGCTG		
RT_DUSP4_F	GGCATCACGGCTCTGTTGAA	DUSP4	
RT_DUSP4_R	TGTCGGCCTTGTGGTTATCT		
RT_FOXA1_F	CTACTACGCAGACACGCAGG	FOXA1	
RT_FOXA1_R	CCGCTCGTAGTCATGGTGTT		
RT_TTC6_F	CACTGATAAGCCGGACTAACG	TTC6	
RT_TTC6_R	ATTTCCCCGTCCAACATAAGC		
