# Supplementary material for: 3D genomic analysis reveals novel enhancer-hijacking caused by complex structural alterations that drive oncogene overexpression
Source: Nat Commun. 2024 Jul 20;15:6130. doi: 10.1038/s41467-024-50387-w (PMC11271278; doi:10.1038/s41467-024-50387-w)
Supplement: Supplementary file 3 — Description of Additional Supplementary Files [file 41467_2024_50387_MOESM3_ESM.pdf]

Description of Additional Supplementary Files

Supplementary Data 1

Included are HAPI genes identified from each cell line in the manuscript. For each HAPI gene, the number of associated enhancer-promoter loops (loops), the summed number of PETs supporting those loops (PETs), the percentage of trans enhancer contribution (Percent_inter), the percentage of abnormal cis enhancer contribution (Percent_intra), and the percentage of normal cis enhancer contribution (Percent_normal) are listed.
