# Supplementary material for: 3D genomic analysis reveals novel enhancer-hijacking caused by complex structural alterations that drive oncogene overexpression
Source: Nat Commun. 2024 Jul 20;15:6130. doi: 10.1038/s41467-024-50387-w (PMC11271278; doi:10.1038/s41467-024-50387-w)
Supplement: Supplementary file 5 — Reporting Summary [file 41467_2024_50387_MOESM5_ESM.pdf]

nature portfolio

Corresponding author(s): Xiaoyang Zhang, Katherine E. Varley

Last updated by author(s): Jun 20, 2024

Reporting Summary

Nature Portfolio wishes to improve the reproducibility of the work that we publish. This form provides structure for consistency and transparency in reporting. For further information on Nature Portfolio policies, see our Editorial Policies and the Editorial Policy Checklist.

Statistics

For all statistical analyses, confirm that the following items are present in the figure legend, table legend, main text, or Methods section.

n/a	Confirmed	
☐	☒	The exact sample size (n) for each experimental group/condition, given as a discrete number and unit of measurement
☐	☒	A statement on whether measurements were taken from distinct samples or whether the same sample was measured repeatedly
☐	☒	The statistical test(s) used AND whether they are one- or two-sided<br>*Only common tests should be described solely by name; describe more complex techniques in the Methods section.*
☒	☐	A description of all covariates tested
☐	☒	A description of any assumptions or corrections, such as tests of normality and adjustment for multiple comparisons
☐	☒	A full description of the statistical parameters including central tendency (e.g. means) or other basic estimates (e.g. regression coefficient) AND variation (e.g. standard deviation) or associated estimates of uncertainty (e.g. confidence intervals)
☐	☒	For null hypothesis testing, the test statistic (e.g. F, t, r) with confidence intervals, effect sizes, degrees of freedom and P value noted<br>*Give P values as exact values whenever suitable.*
☒	☐	For Bayesian analysis, information on the choice of priors and Markov chain Monte Carlo settings
☒	☐	For hierarchical and complex designs, identification of the appropriate level for tests and full reporting of outcomes
☐	☒	Estimates of effect sizes (e.g. Cohen's d, Pearson's r), indicating how they were calculated

Our web collection on statistics for biologists contains articles on many of the points above.

Software and code

Policy information about availability of computer code

Data collection	No softwares were used for data collection

Data analysis

Our own codes for HAPI analysis are accessible at Code Ocean: https://codeocean.com/capsule/2804329, with test examples included.
We also used the following publicly available softwares:
Bowtie2 (2.2.9) for ChIP-seq and RNA-seq reads alignment
BWA-MEM (0.7.17) for WGS reads alignment
HiC-Pro (2.11.1) for HiChIP reads alignment
samtools (1.16) fort sorting and indexing alignments
MACS (2.2.7) for ChIP-seq peak calling
bedtools (2.28.0) for HiChIP and ChIP-seq anchor comparison
RSEM (1.3.0) for quantifying RNA-seq reads
edgeR (3.24.3) for differential gene expression analysis
hichipper (0.7.7) for HiChIP loop calling
gTrack (0.1.0) for HiChIP and ChIP-seq signal presentation
GRIDSS (2.13.2) for calling structural variants from WGS
AmpliconSuite (v1.0.0) for calling complex amplicons and ecDNA from WGS
HOMER (v4.11.1) for super-enhancer calling
Fiji (2.15.0) for analyzing DNA FISH images
Cell Profiler (4.2.1) for analyzing DNA FISH images
deepTools (3.3.0) for virtual 4C signal comparison and presentation

For manuscripts utilizing custom algorithms or software that are central to the research but not yet described in published literature, software must be made available to editors and reviewers. We strongly encourage code deposition in a community repository (e.g. GitHub). See the Nature Portfolio guidelines for submitting code & software for further information.

Data

Policy information about availability of data

All manuscripts must include a data availability statement. This statement should provide the following information, where applicable:

- Accession codes, unique identifiers, or web links for publicly available datasets
- A description of any restrictions on data availability
- For clinical datasets or third party data, please ensure that the statement adheres to our policy

The publicly available H3K27ac HiChIP data used in this study were obtained from the NCBI Gene Expression Omnibus (GEO) at GSE227242 [https://0-www-ncbi-nlm-nih-gov.brum.beds.ac.uk/geo/query/acc.cgi?acc=GSE227242], GSE97585 [https://0-www-ncbi-nlm-nih-gov.brum.beds.ac.uk/geo/query/acc.cgi?acc=GSE97585], GSE157381 [https://0-www-ncbi-nlm-nih-gov.brum.beds.ac.uk/geo/query/acc.cgi?acc=GSE157381], GSE147854 [https://0-www-ncbi-nlm-nih-gov.brum.beds.ac.uk/geo/query/acc.cgi?acc=GSE147854], GSE166232 [https://0-www-ncbi-nlm-nih-gov.brum.beds.ac.uk/geo/query/acc.cgi?acc=GSE166232], GSE151002 [https://0-www-ncbi-nlm-nih-gov.brum.beds.ac.uk/geo/query/acc.cgi?acc=GSE151002], GSE159985 [https://0-www-ncbi-nlm-nih-gov.brum.beds.ac.uk/geo/query/acc.cgi?acc=GSE159985], and GSE188401 [https://0-www-ncbi-nlm-nih-gov.brum.beds.ac.uk/geo/query/acc.cgi?acc=GSE188401] (specific cell lines are described in Supplementary Table 1). The publicly availably ChIP-seq data used in this study were downloaded from GEO at GSE73994 [https://0-www-ncbi-nlm-nih-gov.brum.beds.ac.uk/geo/query/acc.cgi?acc=GSE73994], GSE63109 [https://0-www-ncbi-nlm-nih-gov.brum.beds.ac.uk/geo/query/acc.cgi?acc=GSE63109], GSE69558 [https://0-www-ncbi-nlm-nih-gov.brum.beds.ac.uk/geo/query/acc.cgi?acc=GSE69558], GSE105760 [https://0-www-ncbi-nlm-nih-gov.brum.beds.ac.uk/geo/query/acc.cgi?acc=GSE105760], GSE105627 [https://0-www-ncbi-nlm-nih-gov.brum.beds.ac.uk/geo/query/acc.cgi?acc=GSE105627], GSE85158 [https://0-www-ncbi-nlm-nih-gov.brum.beds.ac.uk/geo/query/acc.cgi?acc=GSE85158], GSE57436 [https://0-www-ncbi-nlm-nih-gov.brum.beds.ac.uk/geo/query/acc.cgi?acc=GSE57436], GSE86743 [https://0-www-ncbi-nlm-nih-gov.brum.beds.ac.uk/geo/query/acc.cgi?acc=GSE86743], GSE73319 [https://0-www-ncbi-nlm-nih-gov.brum.beds.ac.uk/geo/query/acc.cgi?acc=GSE73319], GSE115123 [https://0-www-ncbi-nlm-nih-gov.brum.beds.ac.uk/geo/query/acc.cgi?acc=GSE115123], and GSE159972 [https://0-www-ncbi-nlm-nih-gov.brum.beds.ac.uk/geo/query/acc.cgi?acc=GSE159972] for H3K27ac, and GSE174109 [https://0-www-ncbi-nlm-nih-gov.brum.beds.ac.uk/geo/query/acc.cgi?acc=GSE174109] and GSE94013 [https://0-www-ncbi-nlm-nih-gov.brum.beds.ac.uk/geo/query/acc.cgi?acc=GSE94013] for AR ChIP-seq (specific cell lines are described in Supplementary Table 1). TCGA copy number segment data for all applicable cell lines were downloaded from the Broad Institute Portal (URL: https://data.broadinstitute.org/ccle_legacy_data/dna_copy_number/CCLE_copynumber_2013-12-03.seg.txt). CCLE RNA-seq data were downloaded from the DepMap portal (URL: https://depmap.org/portal/). RNA-seq data for CUTLL1 was downloaded from GSE61999 [https://0-www-ncbi-nlm-nih-gov.brum.beds.ac.uk/geo/query/acc.cgi?acc=GSE61999]. Publicly available WGS data for cell lines were downloaded from NCBI SRA: PRJNA361316 [https://www.ncbi.nlm.nih.gov/sra/?term=PRJNA361316], PRJNA523380 [https://www.ncbi.nlm.nih.gov/sra/?term=PRJNA523380], and PRJNA506071 [https://www.ncbi.nlm.nih.gov/sra/?term=PRJNA506071] (specific cell lines are described in Supplementary Table 1). PCAWG consensus call sets of structural variants in tumor samples were downloaded from the ICGC Data Portal (URL: https://dcc.icgc.org/releases/PCAWG/consensus_sv). Complex amplicon data from PCAWG were downloaded from the AmpliconRepository (https://ampliconrepository.org). Publicly available TCGA ATAC-seq data were downloaded from the UCSC Xena Browser (URL: https://xenabrowser.net/datapages/?dataset=TCGA_ATAC_peak_Log2Counts_dedup_sample&host=https%3A%2F%2Fatacseq.xenahubs.net&removeHub=https%3A%2F%2Fxena.treehouse.gi.ucsc.edu%3A443). The ChIP-seq, HiChIP, and RNA-seq data generated in this study have been deposited to GEO under the series GSE228247 [https://0-www-ncbi-nlm-nih-gov.brum.beds.ac.uk/geo/query/acc.cgi?acc=GSE228247]. The remaining data are available within the Article, Supplementary Information, Supplementary Data, or Source Data file. Source data are provided with this paper.

Research involving human participants, their data, or biological material

Policy information about studies with human participants or human data. See also policy information about sex, gender (identity/presentation), and sexual orientation and race, ethnicity and racism.

Reporting on sex and gender

Use the terms sex (biological attribute) and gender (shaped by social and cultural circumstances) carefully in order to avoid confusing both terms. Indicate if findings apply to only one sex or gender; describe whether sex and gender were considered in study design; whether sex and/or gender was determined based on self-reporting or assigned and methods used.
Provide in the source data disaggregated sex and gender data, where this information has been collected, and if consent has been obtained for sharing of individual-level data; provide overall numbers in this Reporting Summary. Please state if this information has not been collected.
Report sex- and gender-based analyses where performed, justify reasons for lack of sex- and gender-based analysis.

Reporting on race, ethnicity, or [illegible]

Please specify the socially constructed or socially relevant categorization variable(s) used in your manuscript and explain why

Reporting on race, ethnicity, or other socially relevant groupings	*they were used. Please note that such variables should not be used as proxies for other socially constructed/relevant variables (for example, race or ethnicity should not be used as a proxy for socioeconomic status).*<br>*Provide clear definitions of the relevant terms used, how they were provided (by the participants/respondents, the researchers, or third parties), and the method(s) used to classify people into the different categories (e.g. self-report, census or administrative data, social media data, etc.)*<br>*Please provide details about how you controlled for confounding variables in your analyses.*
Population characteristics	*Describe the covariate-relevant population characteristics of the human research participants (e.g. age, genotypic information, past and current diagnosis and treatment categories). If you filled out the behavioural & social sciences study design questions and have nothing to add here, write "See above."*
Recruitment	*Describe how participants were recruited. Outline any potential self-selection bias or other biases that may be present and how these are likely to impact results.*
Ethics oversight	*Identify the organization(s) that approved the study protocol.*

Note that full information on the approval of the study protocol must also be provided in the manuscript.

Field-specific reporting

Please select the one below that is the best fit for your research. If you are not sure, read the appropriate sections before making your selection.

☒ Life sciences ☐ Behavioural & social sciences ☐ Ecological, evolutionary & environmental sciences

For a reference copy of the document with all sections, see nature.com/documents/nr-reporting-summary-flat.pdf

Life sciences study design

All studies must disclose on these points even when the disclosure is negative.

Sample size	Sample size is described in the figure legends of the manuscript. We did not perform any computation to pre-determine sample sizes. We chose the sample sizes based on previous literature, common standards in the field, and experimental feasibility. The results suggest the chosen sample sizes are appropriate because of statistical significance or observed clear distinctions.
Data exclusions	No data was excluded.
Replication	Three biological replicates were performed for molecular and cellular assays that compare different biological conditions. Replicates yielded similar results as shown in the figures.
Randomization	No randomization was applied to the experiments in the study as they were based on in vitro cells grouped by distinct genetic perturbations.
Blinding	Blinding was not applied to the study as the grouping information was necessary for data acquisition and interpretation.

Reporting for specific materials, systems and methods

We require information from authors about some types of materials, experimental systems and methods used in many studies. Here, indicate whether each material, system or method listed is relevant to your study. If you are not sure if a list item applies to your research, read the appropriate section before selecting a response.

Materials & experimental systems

n/a	Involved in the study	
☐	☒	Antibodies
☐	☒	Eukaryotic cell lines
☒	☐	Palaeontology and archaeology
☒	☐	Animals and other organisms
☒	☐	Clinical data
☒	☐	Dual use research of concern
☒	☐	Plants

Methods

n/a	Involved in the study	
☐	☒	ChIP-seq
☒	☐	Flow cytometry
☒	☐	MRI-based neuroimaging

Antibodies

Antibodies used	H3K27ac, Abcam, ab4729, rabbit polyclonal (amount: 4ug/ChIP or 7.5ug/HiChIP)
Validation	H3K27ac (Abcam, ab4729) has been validated for ChIP assays in human cells included in the ENCODE project (PMID: 22955616) and HiChIP assays in human cells (PMID: 28945252)

Eukaryotic cell lines

Policy information about cell lines and Sex and Gender in Research

Cell line source(s)	Prostate cancer cell lines LNCaP, MDAPCA2B, 22Rv1, and VCaP, ER-positive breast cancer cell lines ZR751 and MCF7, lymphoma cell lines REC1 and DOHH2, lung squamous cell line NCIH2170, and small cell lung cancer cell lines NCIH446 and NCIH1876 were obtained from the Cancer Cell Line Encyclopedia (CCLE). The lymphoma cell line CUTLL1 was obtained from Sigma.
Authentication	The identities of the cell lines were verified by either SNP fingerprinting as previously described in the CCLE project (PMID: 22460905) or short tandem repeat analysis through IDEXX.
Mycoplasma contamination	All cell lines were tested negative for mycoplasma using the Lonza MycoAlert kit.
Commonly misidentified lines (See ICLAC register)	No commonly misidentified cell lines were used in the study.

Plants

Seed stocks	*Report on the source of all seed stocks or other plant material used. If applicable, state the seed stock centre and catalogue number. If plant specimens were collected from the field, describe the collection location, date and sampling procedures.*
Novel plant genotypes	*Describe the methods by which all novel plant genotypes were produced. This includes those generated by transgenic approaches, gene editing, chemical/radiation-based mutagenesis and hybridization. For transgenic lines, describe the transformation method, the number of independent lines analyzed and the generation upon which experiments were performed. For gene-edited lines, describe the editor used, the endogenous sequence targeted for editing, the targeting guide RNA sequence (if applicable) and how the editor was applied.*
Authentication	*Describe any authentication procedures for each seed stock used or novel genotype generated. Describe any experiments used to assess the effect of a mutation and, where applicable, how potential secondary effects (e.g. second site T-DNA insertions, mosiacism, off-target gene editing) were examined.*

ChIP-seq

Data deposition

☒ Confirm that both raw and final processed data have been deposited in a public database such as GEO.

☒ Confirm that you have deposited or provided access to graph files (e.g. BED files) for the called peaks.

Data access links *May remain private before publication.*	The ChIP-seq generated in this study have been deposited to GEO under the series GSE228247.
Files in database submission	GSM7115925 LNCaP_NT2_H3K27ac_ChIP<br>GSM7115926 LNCaP_NT1_H3K27ac_ChIP<br>GSM7115927 LNCaP_e3_1_H3K27ac_ChIP<br>GSM7115928 LNCaP_e3_2_H3K27ac_ChIP<br>GSM7115929 LNCaP_e4_1_H3K27ac_ChIP<br>GSM7115930 LNCaP_e4_2_H3K27ac_ChIP
Genome browser session (e.g. UCSC)	Not applicable.

Methodology

Replicates	For assessing ChIP-seq signal after CRISPRi-mediated enhancer repression, we used two separate sgRNAs for each enhancer. We also included two separate non-targeting sgRNAs as negative controls.
Sequencing depth	Please see detailed information of the deposited datasets in GEO (accession #GSE228247).
Antibodies	H3K27ac, Abcam, ab4729, rabbit polyclonal (amount: 4ug/ChIP)
Peak calling parameters	macs2 callpeak -t bam_file -f BAM -n name -B --SPMR --broad
Data quality	We used q-value of 0.05 to select significant ChIP-seq peaks.
Software	Bowtie2 (2.2.9) for ChIP-seq read alignment<br>Samtools (1.16) for sorting and indexing alignments<br>MACS2 (2.2.7) for ChIP-seq peak calling<br>bedtools (2.28.0) for ChIP-seq peaks comparison<br>Homer (v4.11.1) for super-enhancer identification
